# Autologous platelet concentrates in alveolar ridge preservation: A systematic review with meta‐analyses

**DOI:** 10.1111/prd.12609

**Published:** 2024-09-30

**Authors:** Sayed Ahmad Manoetjer Siawasch, Jize Yu, Ana B. Castro, Rutger Dhondt, Wim Teughels, Andy Temmerman, Marc Quirynen

**Affiliations:** ^1^ Department of Oral Health Sciences, Periodontology, KU Leuven & Dentistry University Hospitals Leuven Leuven Belgium

**Keywords:** alveolar ridge preservation, autologous platelet concentrates, extraction socket, leukocyte‐ and platelet‐rich fibrin, plasma rich in growth factor, platelet rich fibrin, platelet rich plasma.

## Abstract

In order to evaluate the therapeutic advantages of various autologous platelet concentrates (APC) as a single biomaterial during alveolar ridge preservation (ARP), a systematic review with meta‐analyses was conducted. PubMed, EMBASE, Web of Science, and Scopus were screened for randomized controlled trials (RCTs) that were released prior to 2024. The selected papers compared an APC with either unassisted healing (blood clot) or another biomaterial during ARP (third molars were not included). The outcome parameters included alveolar bone dimension alterations, soft tissue healing, and post‐op pain intensity. The search yielded 35 papers (33 studies), one applying platelet‐rich plasma (PRP), six using plasma rich in growth factors (PRGF), and 28 using leukocyte‐ and platelet‐rich fibrin (L‐PRF). These studies showed a large heterogeneity (e.g., outcome parameters, timing, surgical approach, and inclusion criteria), which hindered drawing strong conclusions. In most studies, however, ARP with PRP, PRGF, and L‐PRF alone produced faster soft tissue healing, less post‐extraction pain, less alveolar ridge resorption, more socket bone fill, and a higher bone density when compared to unassisted (spontaneous) healing. The ultimate benefit appears to be significantly influenced by the surgical approach. Limited literature exists comparing APC with other biomaterials for ARP, resulting in inconclusive data. APC application for ARP is a promising strategy to improve soft and hard tissue healing and reduce post‐extraction pain.

## 1 INTRODUCTION

After tooth extraction, alveolar atrophy occurs in both horizontal and vertical dimensions due to a combination of soft and hard tissue changes.^1^ Tissue resorption is more prominent on the buccal than on the lingual/palatal site because the buccal bone wall is thinner and the crestal part of the buccal bone is primarily occupied with bundle bone, a tooth‐dependent tissue that resorbs fast after tooth extraction.^2^ Unlike the moderate changes in bone height (approximately 1.7 mm), the horizontal reduction (approximately 3–4 mm) can go up to 60% of the initial alveolar ridge width. A systematic review with meta‐analyses reported pooled estimates for mean horizontal and mid‐facial vertical ridge resorption, when assessed clinically (non‐molar sites), of 2.7 mm (95% CI: 2.4–3.1) and 1.7 mm (95% CI: 1.3–2.1), and when assessed radiographically (non‐molar sites), of 2.5 mm (95% CI: 2.0–3.1) and 1.7 mm (95% CI: 0.4–2.9), respectively.^3^ Approximately two‐thirds of this reduction occurred in the first 3 months after tooth extraction.^3^, ^4^, ^5^, ^6^

Factors that influence this alveolar ridge atrophy include:

- the location of the extracted tooth (front vs. premolar vs. molar, maxilla vs. mandible),
- the number of extracted teeth (single vs. multiple neighboring sites),
- extraction technique (atraumatic vs. traumatic; flap vs. flapless; integrity of the buccal bone plate),
- bone phenotype (buccal bone plate thickness <1.0 mm vs. ≥1.0 vs. ≥2.0 mm),
- prosthetic reconstruction during socket healing (fixed vs. removable),
- presence of a pathology that could damage the bone before the extraction (e.g., extended apical lesion or severe periodontal breakdown).^3^, ^5^, ^6^, ^7^, ^8^, ^9^, ^10^, ^11^, ^12^

In the early 70s, root banking was described as a technique to preserve the alveolar bone. The idea behind this method is to decapitate and remove the crown of the tooth while leaving the root with its periodontal unit in situ. There are certain drawbacks to this procedure. The tooth needs to be endodontically treated, which is expensive and time‐consuming. Furthermore, good coronal sealing is required to prevent caries formation and recontamination of the root canals. And the patient also needs to maintain good oral hygiene to prevent plaque accumulation and periodontal disease. Besides, the primary goal of this technique was to prevent bone resorption and to maintain a good fit of the removable prosthesis.^13^, ^14^

Alveolar ridge preservation (ARP) is another technique to prevent or minimize the changes in soft and hard tissues associated with the natural healing processes after tooth extraction. As such, ARP can facilitate optimal implant placement if immediate implant placement is not possible or preserves the alveolar dimensions at the pontic side in case of a bridge (on natural teeth or dental implants).^10^, ^15^, ^16^, ^17^ Furthermore, ARP has the potential to promote healing and reduce post‐extraction complications.^18^, ^19^, ^20^ Three different approaches for ARP can be utilized depending on the clinical indications:

- sealing the extraction socket without adding any grafting material inside the socket,
- grafting the extraction socket with a bone substitute without sealing the socket,
- or grafting the extraction socket with a bone substitute, followed by sealing of the socket.^16^

In case of socket grafting, different bone substitutes like allograft, dentin graft, calcium phosphate, hydroxyapatite, and biologics such as autologous platelet concentrates (APC) or enamel matrix derivatives have been successfully used.^9^, ^20^, ^21^, ^22^ Furthermore, a variety of materials can be used for socket sealing, including free gingival graft, connective tissue graft, collagen membrane, PTFE membrane, and APC membrane. Depending on the chosen biomaterials, ARP can be performed with or without primary closure. While autogenous bone can be used at a lower cost and heal more quickly, its rapid resorption and donor side postoperative morbidity continue to be drawbacks.^23^, ^24^, ^25^

The gold standard for ARP has been described as applying a xenograft for socket grafting along with a barrier membrane or a free gingival punch for socket sealing.^17^, ^26^ However, a recent systematic review found no statistically significant differences between the barriers and/or grafting materials used for ARP.^27^

A Cochrane review by Atieh et al.^27^ including six studies, 184 participants, and 201 extraction sites compared unassisted socket healing with the use of a xenograft and barrier membrane for ARP and reported a significant reduction in alveolar width resorption (mean difference: ‐1.2 mm, 95% CI ‐1.8–‐0.5; *p* = 0.0003) and in alveolar height resorption (mean difference: ‐1.4 mm, 95% CI ‐2.0–‐0.7; *p* < 0.0001) in favor of the ARP, however, without a significant difference for the need of additional augmentation (RR 0.68, 95% CI 0.29–1.62; *p* = 0.39; 4 studies; 154 participants; 156 extraction sites).^28^, ^29^, ^30^, ^31^, ^32^, ^33^

Couso‐Queiruga et al. reported that additional bone grafting at delayed implant placement was less needed but not unavoidable after ARP, especially at sites exhibiting thin facial bone (≤1 mm) at baseline.^10^, ^11^ Short‐term results also reveal no differences in survival or success rate of dental implants placed after ARP in comparison to unassisted socket healing.^27^, ^34^ However, other studies seem to indicate that sites reconstructed with xenogenic material might be more vulnerable to early implant failure, perhaps because of the slow resorption of the xenograft.^35^, ^36^

This systematic review aimed to explore the clinical benefits (e.g., reduction in alveolar bone resorption, bone fill in socket, soft tissue healing, post‐extraction pain) after applying an APC when compared to unassisted healing or to another biomaterial during alveolar ridge preservation. The review makes a distinction between the different APC [PRP, PRGF, and L‐PRF family (the latter including the original L‐PRF, A‐PRF, A‐PRF+, T‐PRF, H‐PRF, and CGF)] and looks into details like the variation in surgical approach (flap vs. flapless, APC to fill or to fill and cover the socket), the amount of APC matrices applied, the tooth type and position, the reason for tooth extraction, etc.

## 2 MATERIALS AND METHODS

### 2.1 Focused question

What is the clinical benefit of applying an APC in a non‐third molar extraction socket compared to unassisted healing or to the use of another biomaterial during ARP, based on data from randomized controlled trials?

### 2.2 PICO strategy

*Population*: Systemically healthy humans in need of ARP following tooth extraction (non‐third molars).
*Intervention*: Tooth extraction followed by the application of an APC [platelet‐rich plasma (PRP), plasma rich in growth factor (PRGF), or leukocyte‐ and platelet‐rich fibrin (L‐PRF)] alone in the extraction socket (without the use of other biomaterials).
*Comparison*: Tooth extraction with unassisted healing (blood clot only) or an ARP with another biomaterial than APC.
*Outcomes*: Hard tissue healing, soft tissue healing (e.g., healing index, socket closure), and post‐extraction pain.

### 2.3 Primary and secondary outcomes

Alveolar ridge dimensional changes after tooth extraction were selected as primary variables, including horizontal alveolar width, vertical alveolar crest height, proportion of socket fill, and bone quality. Soft tissue healing and postoperative pain (VAS score, analgesic consumption) were selected as secondary parameters.

### 2.4 Inclusion and exclusion criteria

Only randomized controlled trials (RCTs) with at least 10 systemically healthy humans were considered, either with a split‐mouth or a parallel design. Both short‐term studies (e.g., 2–3 weeks, often reporting on soft tissue healing, post‐extraction pain, socket closure) as well as long‐term studies (important for the alveolar ridge dimension changes) were included. Studies on third molar extractions; studies realized in patients undergoing head and neck radiotherapy; patients with bone diseases; patients with immune‐systemic diseases or uncontrolled diabetes; studies investigating the combination of APC with other materials were all excluded. Articles representing reviews or based on prospective and retrospective cohort studies and case series, studies including fewer than five sockets per group, in vitro studies, animal studies, and studies not published in English were also excluded.

### 2.5 Search methodology

Detailed search strategies followed by manual searching were conducted through the following electronic databases: PubMed (MEDLINE), EMBASE, Web of Science, and Scopus. Only papers published in English until December 2023 were selected.

The search was performed using the following search terms: ((extraction[Title/Abstract]) OR (extraction socket[Title/Abstract]) OR (post‐extraction[Title/Abstract]) OR (post extraction[Title/Abstract]) OR (postextraction[Title/Abstract]) OR (unassisted healing[Title/Abstract]) OR (tooth socket[Title/Abstract]) OR (alveolar defect[Title/Abstract]) OR (alveolar ridge[Title/Abstract]) OR (alveolar ridge preservation[Title/Abstract]) OR (alveolar ridge reconstruction[Title/Abstract]) OR (alveolar ridge augmentation[Title/Abstract]) OR (alveolar ridge resorption[Title/Abstract]) OR (alveolar bone[Title/Abstract]) OR (alveolar socket[Title/Abstract]) OR (socket management[Title/Abstract]) OR (socket preservation[Title/Abstract]) OR (socket seal[Title/Abstract]) OR (socket grafting[Title/Abstract])) AND ((healing[Title/Abstract]) OR (socket healing[Title/Abstract]) OR (bone healing[Title/Abstract]) OR (socket fill[Title/Abstract]) OR (socket regeneration[Title/Abstract]) OR (bone regeneration[Title/Abstract]) OR (bony regeneration[Title/Abstract]) OR (osseous regeneration[Title/Abstract]) OR (alveolar ridge resorption[Title/Abstract]) OR (alveolar bone repair[Title/Abstract]) OR (trabecular bone[Title/Abstract]) OR (bone resorption[Title/Abstract]) OR (bone proportion[Title/Abstract]) OR (mineralised tissue[Title/Abstract]) OR (mineralized tissue[Title/Abstract]) OR (vital bone formation[Title/Abstract]) OR (soft tissue healing[Title/Abstract]) OR (soft tissue closure[Title/Abstract])) AND ((PRGF[Title/Abstract]) OR (plasma rich in growth factor[Title/Abstract]) OR (PRP[Title/Abstract]) OR (platelet rich plasma[Title/Abstract]) OR (PRF[Title/Abstract]) OR (platelet rich fibrin[Title/Abstract]) OR (L‐PRF[Title/Abstract]) OR (leukocytes and platelet rich fibrin[Title/Abstract]) OR (CGF[Title/Abstract]) OR (concentrated growth factor[Title/Abstract]) OR (platelet[Title/Abstract]) OR (platelet concentrate[Title/Abstract]) OR (blood concentrate[Title/Abstract]) OR (autologous platelet[Title/Abstract])) NOT ((sinus floor[Title/Abstract]) OR (guided bone regeneration [Title/Abstract]) OR (autotransplantation [Title/Abstract]) OR (3rd molar[Title/Abstract]) OR (third molar[Title/Abstract]) OR (third molars[Title/Abstract]) OR (wisdom tooth[Title/Abstract]) OR (wisdom teeth[Title/Abstract]) OR (review[Title/Abstract])).

The reference lists of included papers and systematic reviews were evaluated (cross‐referenced) to identify other studies for potential inclusion.

### 2.6 Study selection

Two independent reviewing authors (S.A.M.S. and J.Y.) carried out the search and screening process. Following the examination of titles and abstracts, full papers were selected for careful reading when matching the eligibility criteria for data extraction. If disagreements arose among the reviewing authors, a discussion ensued.

### 2.7 Data extraction and synthesis

Two reviewers (S.A.M.S. and M.Q.) independently extracted the data from the included studies. The following data were considered: authors, study design (split‐mouth or parallel), follow‐up period, number of subjects, age (range or mean), gender, number/proportion of smokers, number of extracted teeth (single or multiple neighboring sites), tooth location (upper jaw/lower jaw; incisor/canine/premolar/molar), reason for tooth extraction, surgical approach (flap/flapless, healing by primary/secondary intention), centrifugation protocol (system, device, and settings), mode of APC application [socket fill (with a gel/clot/membrane), socket sealing, or both], and the aforementioned outcome parameters.

To better understand the advantageous impact of APC during ARP, a distinction was made between platelet‐rich plasma (PRP), plasma rich in growth factors (PRGF), and leukocyte‐ and platelet‐rich fibrin [the L‐PRF family, comprising the original L‐PRF protocol, as well as advanced PRF (A‐PRF), advanced PRF plus (A‐PRF+), titanium PRF (T‐PRF), horizontal PRF (H‐PRF), and concentrated growth factors (CGF)]. Moreover, a separate analysis was conducted to compare the following treatment options:

- APC as a single biomaterial in comparison to unassisted healing (natural healing with an unmodified blood clot),
- APC as a single biomaterial in comparison to other biomaterials used for socket grafting and/or socket sealing.

### 2.8 Meta‐analyses

The most commonly used parameters (reduction of horizontal and vertical alveolar bone dimensions and percentage of socket fill) were the focus of these meta‐analyses. The horizontal alveolar bone width data for measurement at the crest and those 2–3 mm below the crest were examined separately in order to reduce variability. For the vertical height, the buccal bone plate was considered, enabling the incorporation of the most studies. Only data (in millimeters) from CBCT images, taken both immediately after tooth extraction and several months later, were taken into account for these studies. Separate analyses were conducted for the PRP, PRGF, and L‐PRF family data. Publications with a high risk of bias or those without standard deviations were not included.

### 2.9 Risk of bias

Using the RoB 2 tool from the Cochrane Handbook for Systematic Reviews of Interventions,^37^, ^38^, ^39^ two authors (J.Y. and M.Q.) assessed the risk of bias by addressing the following five domains:

- Domain 1: bias arising from the randomization process (especially randomization and site allocation),
- Domain 2: bias due to deviations from intended interventions (especially blinding of participants, surgeons, and personnel),
- Domain 3: bias in measurement of the outcome (especially blinding towards outcome assessment/outcome assessors),
- Domain 4: bias due to missing outcome data (data available for all participants),
- Domain 5: bias in the selection of the reported result (data analyzed in accordance with a prespecified analysis plan).

None of the reviewers was blinded to the names of the authors, institutions, journals, or results of a study. Both reviewers evaluated the possibility of bias separately from one another. When there was a disagreement, a third review author (S.A.M.S.) helped to achieve a consensus. Using the following approach, the overall risk of bias for each individual study was estimated:

- Low risk of bias: (plausible bias unlikely to seriously alter the results) if all criteria were met.
- Some concerns: (plausible bias that raises some doubt about the results) if one or more criteria were partly met or if there was insufficient information to know if they were met.
- High risk of bias: (plausible bias that seriously weakens confidence in the results) if one or more criteria were not met as described in the Cochrane Handbook for Systematic Reviews of Interventions or when four or five criteria had some concerns.^37^, ^38^, ^39^

## 3 RESULTS

### 3.1 Study selection

**FIGURE 1:**
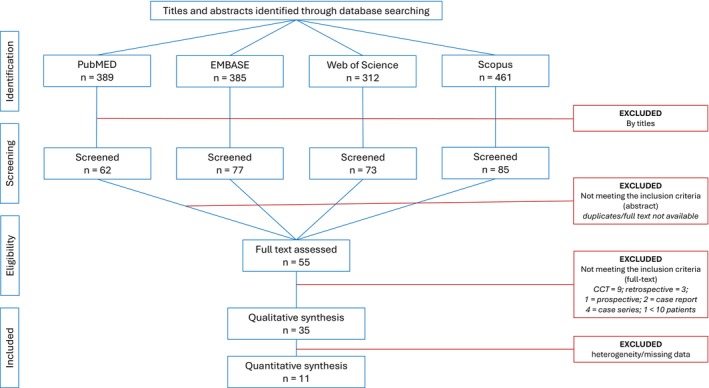
Flow chart of the search strategy.

A total of 1547 papers were identified via the search strategy. After the removal of studies that did not fulfill the inclusion criteria and/or were in duplicate, 55 papers remained. Following full‐text screening, another 20 papers were excluded, resulting in a final selection of 35 papers (33 studies) appropriate for qualitative analysis, of which 11 could be used for a quantitative analysis (Figure 1).

### 3.2 Heterogeneity

The studies showed a very large heterogeneity, which made it difficult to perform quantitative analyses. The most notable differences across the studies were:

- distinct study designs: split‐mouth vs. parallel groups;
- distinct types of APC: PRP, PRGF and L‐PRF;
- distinct centrifugation protocols for the preparation of L‐PRF (concept similar to the original L‐PRF protocol vs. A‐PRF protocol vs. T‐PRF vs. CGF protocol);
- distinct ways to apply APC, such as gel, fibrin clot, and membrane;
- different ridge preservation strategies: solely socket grafting vs. solely socket sealing vs. a combination of socket grafting and sealing;
- different locations of treated teeth (upper and/or lower jaw, incisors and/or canines, and/or premolars, and/or molars);
- different reasons for tooth extraction (bone loss, caries, cracket tooth, endodontic pathology, implant treatment, periodontal disease, occlusal interference, orthodontics, residual root, root fracture, root resorption, tooth fracture, unfavorable prosthetic support, unrestorable);
- large variety in parameters, especially ´how´ and ´where´ the parameters were measured, and a considerable percentage of papers not mentioning the standard deviations.

### 3.3 Risk of bias

**FIGURE 2:**
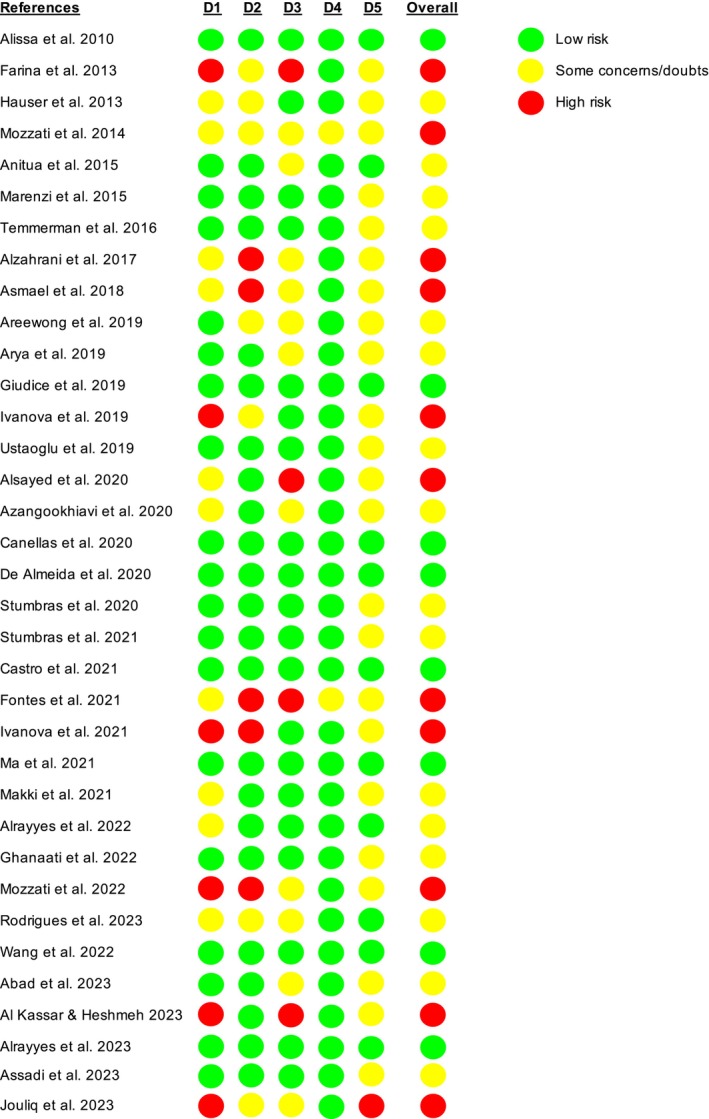
Risk for bias assessment for each individual study considering five domains. D1: Randomization/allocation, allocation concealment, difference in baseline data caused by patient selection; D2: Blinding of participants, surgeons, and personnel; D3: Blinding towards outcome assessment/outcome assessors; D4: Incomplete outcome data; D5: Incomplete outcome data.

**FIGURE 3:**
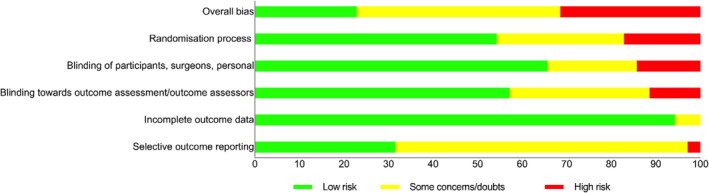
Overall risk of bias graph (authors' judgments) about each risk of bias item presented as percentages across all included studies.

Eight studies were found to have an overall low risk of bias, 16 to have a moderate risk, and the other 11 to have a high risk. The procedures of blinding (participants, surgeons, and staff) and randomization were the domains where high risk was most prevalent (Figures 2 and 3, Tables 1 and 2).

**TABLE 1:** Soft and hard tissue data from RCTs comparing unassisted socket healing (blood clot) with alveolar ridge preservation using an APC as the sole biomaterial.

Article	Study	Subjects	Extraction	Centrifugation	Treatment	Outcome parameters
	Design	Gender	Single/multiple	Device	flap/flapless; PrH/SeH	Data in bold = statistically significant
	Follow‐up	Age	Tooth location: UJ/LJ; I/C/Pr/Mo	rpm or g/min	C = control	Pain/soft tissue healing
Overall bias	Bias risk/domain	% Smokers	Reason for extraction	Coagulation factors	T = test	Hard tissue healing (alveolar bone resorption = ABR)
***A. PRP***						
Alissa et al., 2010^40^ L	RCT parallel 3 m L/L/L/L/L	*n* = 21 mean age = 30y 52% smokers	1 socket/patient single sites UJ & LJ; I/C/Pr/Mo **Ca**, En	IEC Model 3.200 rpm/12′ *thrombin*	Flap/PrH 9 C: blood clot 12 T: PRP gel *to fill*	T gave: **< pain first 3 days, < analgesic consumption first 2 days** **improved soft tissue healing** (*HI index Landry* et al., *1988*) **> trabecular bone volume** (*43* vs. *32%*) = trabecular bone architecture
***B. PRGF***						
Farina et al., 2013^41^ **H**	RCT parallel 10 w H/S/H/L/S	F = 15/M = 13 mean age = 55y 18% smokers	1–2 socket(s)/patient single sites UJ & LJ; I/C/Pr/Mo **Rf**, Im, Ca, En	PRGF System 580 *g*/8′ *calcium chloride*	Flapless/SeH 18 C: blood clot 18 T: PRGF gel *to fill*	T gave: = relative proportion of bone (radiography and histomorphometry)
Mozzati et al., 2014^42^ **H**	RCT split‐mouth 3 w S/S/S/S/S	F = 22/M = 12 mean age = 63y 41% smokers	2 sockets/patient single sites NR; NR **PD**/Ca	NR 460 *g*/8′ *calcium chloride*	Flapless/SeH 34 C: blood clot 34 T: PRGF gel *to fill & cover with fibrin membrane*	T gave: = pain, = post‐surgical complications **improved softtissue healing at days 3, 7, and 14** (*Modified HI index Masse* et al., *1993*) **< residual socket volume at d 3** (*0.2* vs. *0.3*) **and 7** (*0.07* vs. *0.1*)
Anitua et al., 2015^43^ S	RCT parallel 10–12 w L/L/S/L/ L	F = 31/M = 29 mean age = 55y 32% smokers	1 socket/patient single sites LJ; Mo **En**, BL, Ur, Oi, En	BTI System 580 *g*/8′ *calcium chloride*	Flapless/SeH 24 C: blood clot 36 T: PRGF clot *to fill & cover with fibrin membrane*	T gave: **< pain at days 3 and 7, < inflammation at days 3 and 7** **improved soft tissue healing at days 3**, **7, 15** (*HI index Landry* et al., *1988*) **> keratinized gingival thickness** (*0.4* vs. *0.3 mm*) **> bone fill** (*97* vs. *46%*) **> regenerated volume** (*97* vs. *75%*) **> bone density** (*HU: 450* vs. *318*) **higher relative proportion of bone** (*63%* vs. *36%*)
Arya et al., 2019^44^ S	RCT split‐mouth 13 w L/L/S/L/S	F = 7/M = 13 mean age = 24y smokers = NR	2 sockets/patient single sites LJ; Pr/Mo NR	NR NR *calcium chloride*	Flapless/SeH 20 C: blood clot 20 T: PRGF gel *to fill & cover with fibrin membrane*	T gave: **> bone density** (*648* vs. *500 HU*)
Stumbras et al., 2020^45^ *2 of 4 arms* S	RCT parallel 3 m L/L/L/L/S	F = 26/M = 14 mean age = 49y 12% smokers	1 socket/patient single sites LJ; I/C **Ca**, Rf, En, PD	BTI System NR *calcium chloride*	Flapless/SeH 10 C: blood clot 10 T: PRGF clot *to fill & cover with fibrin membrane*	T gave: **higher relative proportion of bone** (*76%* vs. *46%*) **lower relative proportion of non‐mineralized tissue** (*24%* vs. *54%*)
Stumbras et al., 2021^46^ *2 of 4 arms* S	Same	Same	Same	Same	Same	T gave: = horizontal ABR: HW^1^ (*1.3* vs. *1.6 mm*), HW^3^ (*0.2* vs. *0.8 mm*), HW^5^ (*0.0* vs. *0.8 mm*) = vertical ABR (*0.5* vs. *0.9 mm*)
***C. L‐PRF family***						
Hauser et al., 2013^53^ S	RCT parallel 8 w S/S/L/L/S	F = 14/M = 9 mean age = 47y smokers = NR	1 socket/patient single sites UJ & LJ; Pr Ca, En, PD, Rf *	NR 2700 rpm/12′	Flap/PrH; flapless/SeH 8 C: blood clot 9 T_1_: 4 L‐PRF m *flapless* 6 T_2_: 4 L‐PRF m *flap* *both to fill & to cover*	T_1_ gave: = microarchitecture of newly formed trabecular bone vs. C and T_2_ **> intrinsic mechanical properties of new bone vs. to C and T**_**2**_ **< loss of the alveolar width compared to C** (*0.1* vs. *0.4 mm*) and T_2_ (*0.4 mm*)
Marenzi et al., 2015^47^ S	RCT split‐mouth 3 w L/L/L/L/S	F = 17/M = 9 mean age = 53y some light smokers	2–8 sockets/patient multiple sites NR; C/Pr/Mo En, Or, Rer, Tf, Ur *	Intra‐Spin 2700 rpm/12′	Flapless/SeH 54 C: blood clot 54 T: >1 L‐PRF m *to fill*	T gave: **< post‐extraction pain at 24, 48 and 72 h** **improved soft tissue healing at d 14 and 21** (*Modified HI from Mozatti* et al., *2014*)
Temmerman et al., 2016^54^ S	RCT split‐mouth 3 m L/L/L/L/S	F = 7/M = 15 mean age = 54y 0% smokers	2 sockets/patient single sites UJ & LJ; I/C/Pr NR	IntraSpin 2700 rpm/12′	Flapless/SeH 22 C: blood clot 22 T: L‐PRF m, 2–5 *to fill &* 2–3 *to cover*	T gave: **< pain at days 3, 4 and 5** **< vertical height changes (buccal site)** (*0.5* vs. *1.5 mm*) **< total ABR at 1 mm below the crest** (*23%* vs. *52%*) **> socket fill** (*95%* vs. *63%*)
Alzahrani et al., 2017^55^ **H**	RCT parallel 8 w S/H/S/L/S	F = 15/M = 9 mean age = 38y 0% smokers	1 socket/patient single sites NR; NR Ca, En, PD, Rf *	HERMLE 400 *g*/12′	Flapless/SeH 12 C: blood clot 12 T: 2 L‐PRF m *to fill*	T gave: **< less horizontal ABR:** **w 1** (*0.2* vs. *0.5 mm*)**, w 4** (*0.6* vs. *1.4 mm*), **w 8** (*1.0* vs. *1.9 mm*) **> radiographic bone fill:** **w 1** (*74%* vs. *69%*)**, 4** (*82%* vs. *74%*) **and 8** (*89%* vs. *80%*)
Asmael et al., 2018^48^ **H**	RCT split‐mouth 3 w S/H/S/L/S	F = 0/M = 20 mean age = 44y 100% smokers	2 sockets/patient single sites NR; NR PD excl	Xiangtian 3000 rpm/10′	Flapless/SeH 20 C: blood clot 20 T: 1 L‐PRF cl *to fill*	T gave: = pain level, **less inflammation** **improved soft tissue healing at d 7** (*HI index Landry* et al., *1988*) = percentage of epithelialization (*53%* vs. *51%*) at 1 w
Areewong et al., 2019^56^ S	RCT parallel 8 w L/S/S/L/S	*n* = 33 mean age = 51y 6% smokers	1–2 socket(s)/patient single sites UJ & LJ; I/C/Pr En, Tf, Ur *	IntraSpin 2700 rpm/12′	Flapless/SeH 18 C: blood clot 18 T: 1 L‐PRF pl *to fill*	T gave: = relative proportion of bone (*histology: 31%* vs. *26%*)
Giudice et al., 2019^49^ *3 of 4 arms* L	RCT split‐mouth 2 w L/L/L/L/L	F = 12/M = 28 mean age = 61y 38% smokers	4 sockets/patient with antiplatelets single sites NR; I/C/Pr/Mo NR	IntraSpin 2700 rpm/18′	Flap or flapless/SeH 40 C: blood clot 40 T_1_: 2 L‐PRF pl *to fill* 40 T_2_: 2 A‐PRF pl *to fill*	T_1,2_ gave: = soft tissue healing (w1, w2) **< post‐extraction bleeding**
Ivanova et al., 2019^57^ *2 of 3 arms* **H**	RCT parallel 4 m H/S/L/L/S	F = 31/M = 29 mean age = 42y some light smokers	1–2 socket(s)/patient single sites NR; NR NR	PRF DUO 1300 rpm/8′	Flap/PrH 12 C: blood clot 23 T: 2 L‐PRF m *to fill*	T gave: **< vertical ABR** (*0.9* vs. *1.4 mm*) **< horizontal ABR** (*1.5* vs. *2.4 mm*) **higher relative proportion of vital bone** (*61* vs. *37%*) **lower relative proportion of connective tissue** (*29* vs. *54%*)
Ustaoglu et al., 2020^58^ S	RCT parallel 3 m L/L/L/L/S	F = 29/M = 28 mean age = 35y 0% smokers	1 socket/patient single sites NR; I/C/Pr Ca, En, PD, Rf *	L‐PRF: IntraSpin 2700 rpm/12′ T‐PRF: titanium tubes 2800 rpm/12′	Flapless/SeH 19 C: blood clot 19 T_1_: 2–3 L‐PRF m *to fill* 19 T_2_: 2–3 T‐PRF m *to fill*	T_1,2_ gave: **< pain d1,** = consumption of analgesic = Landry Wound Healing Indexes **> complete wound epithelialization** **at 1st w** (*T*_*1*_ *: 55* vs. *T*_*2*_ *: 70* vs. *C: 10%*) **at 2nd w** (*T*_*1*_ *: 100* vs. *T*_*2*_ *: 100* vs. *C: 41%*) **> fractal values after 3 m (T**_**1**_ **: 1.33 vs. T**_**2**_ **: 1.40 vs. C: 1.24)**
Alsayed et al. 2020^59^ **H**	RCT split‐mouth 6 m S/L/H/L/S	F = 9/M = 11 age = 24‐49y 0% smokers	2 sockets/patient single sites NR; NR BL, PD *	NR 3000 rpm/10′	Flapless/SeH 20 C: blood clot 20 T: 2 L‐PRF cl *to fill*	T gave: **< vertical ABR after 6 m** (*0.8* vs. *1.8 mm*) **< vertical socket height reduction after 6 m** (*0.7* vs. *1.7 mm*) **< horizontal ABR after 6 m** (*0.6* vs. *0.9 mm*) = bone density after 6 m (575 vs. 698)
Canellas et al., 2020^60^ L	RCT parallel 3 m L/L/L/L/L	F = 27/M = 21 mean age = 45y 0% smokers	1 socket/patient single sites UJ & LJ; I/C/Pr Ca, En, Rf, Ups *	IntraSpin 408 *g*/12′	Flapless/SeH 24 C: blood clot 24 T: L‐PRF, 4 *pL to fill &* 2 *m to cover*	T gave: **< horizontal ABR at 1 and 3 mm below the crest** (*0.9* vs. *2.3 mm*, *0.9* vs. *1.7 mm*) **< vertical ABR at buccal side** (*0.7* vs. *1.4 mm*) **> new bone formation** (*191* vs. *45 mm* ^*3*^)
De Almeida et al., 2020^50^ L	RCT parallel 2 w L/L/L/L/L	F = 19/M = 13 mean age = 37y 0% smokers	1 socket/patient single sites UJ & LJ; Pr/Mo **PD**, Ca, Tf, Rf	IntraSpin 708 *g*/12′	Flapless/SeH 16 C: blood clot 16 T: 2 L‐PRF m *to fill*	T gave: **< postoperative pain, < consumption of analgesic** **improved soft tissue healing at day 7** (*HI index Landry* et al., *1988*)
Castro et al., 2021^61^ *3 arms* L	RCT split‐mouth 3 m L/L/L/L/L	F = 15/M = 6 mean age = 64y 14% smokers	3 sockets/patient multiple sites UJ; I/C NR	IntraSpin 2700 rpm/12′	Flapless/SeH 21 C: blood clot 21 T_1_: L‐PRF m 21 T_2_: A‐PRF m *both* 2–3 *to fill &* 1–2 *to cover*	T_1,2_ gave: = changes in horizontal ABR (*1.6* vs. *1.7 mm*) = mean vertical ABR (*0.2* vs. *0.2 mm*) **> socket fill** (*85%* vs. *68%*) **higher relative proportion of bone** (*48%* vs. *35%*)
Fontes et al., 2021^62^ *2 of 3 arms* **H**	RCT parallel 6 m S/H/H/S/S	F = 8/M = 7 mean age = 47y 0% smokers	1 socket/patient single sites UJ; I/C NR	Centribio 400 *g*/12′	Flapless/SeH 5 C: blood clot 5 T: 1 PRF pl *to fill*	T gave: **higher relative proportion of bone** (*54%* vs. *41%*) = osteocalcin expression in bone cores
Ivanova et al., 2021^63^ 2 of 3 arms **H**	RCT parallel 4 m H/H/L/L/S	F = 45/M = 45 mean age = 42y some light smokers	1 socket/patient single sites UJ & LJ; I/C/Pr/Mo PD, Rf, Rr, Tf *	NR NR	Flap/PrH 30 C: blood clot 30 T: 1 L‐PRF m *to fill*	T gave: **higher relative proportion of bone** (*61%* vs. *39%*) **lower relative proportion of connective tissue** (*30%* vs. *52%*)
Ma et al., 2021^64^ L	RCT parallel 3 m L/L/L/L/L	F = 18/M = 28 mean age = 44y some light smokers	1 socket/patient single sites UJ & LJ; Pr/Mo PD excl	Medifuge # rpm for #′	Flapless/SeH 23 C: blood clot 23 T: CGF cl, 1 *to fill &* 1 *to cover*	T gave: **improved soft tissue healing at d 10** (*Modified version of the Masse healing index 1993*) **< vertical ABR** (*0.6* vs. *1.5 mm*) **< horizontal ABR 1 mm apical to crest** (*1.4* vs. *3.1 mm*) **> bone mineral density** (*1121* vs. *936 mg/cm* ^*3*^) **higher relative proportion of bone** (*0.6* vs. *0.5*) **> new trabecular bone** (*4.0* vs. *3.5*)
Makki et al., 2021^51^ S	RCT parallel 2 w S/L/L/L/S	F = 31/M = 29 age = 18‐60y smokers=NR	1 socket/patient single sites UJ & LJ; Pr/Mo Ca, PD *	L‐PRF: Medifuge 2700 rpm/12′ A‐PRF Medifuge 1500 rpm/14′	Flap or flapless/PrH or SeH 20 C: blood clot 20 T_1_: 1 L‐PRF m to *fill* 20 T_2_: 1 A‐PRF m to *fill*	T_1,2_ gave: **< pain first 2 d** **< consumption analgesics at 6 h, 18 h** **improved soft tissue healing: w 1, 2** (*Landry Wound Healing Index*)
Ghanaati et al., 2022^65^ S	RCT parallel 3 m L/L/L/L/S	NR age≥18y smokers=NR	NR NR UJ & LJ; Pr/Mo Im, PD excl, Rf excl	DUO 1200 rpm/8′	Flapless/SeH 31 C: blood clot 33 T: 1 L‐PRF pl/root *to fill*	T gave: = pain first 7 d **< remaining wound size after d 7–10**
Mozzati et al., 2022^52^ **H**	RCT split‐mouth 3 w H/H/S/L/S	F = 42/M = 35 mean age = 57y 14% smokers	2 sockets/patient single sites UJ & LJ; Pr/Mo En, PD, Tf, Ur *	Medifuge # rpm for #′	Flapless/SeH 77 C: blood clot 77 T: 1 CGF cl *to fill*	T gave: **< pain the first 5 days** **improved soft tissue healing at d 7** (*HI index Landry* et al., *1988*) **> socket closure at d 14, 21**
Rodrigues et al., 2023^66^ *2 of 4 arms* S	RCT parallel 4 m S/S/S/L/L	F = 11/M = 9 mean age = 42y(T); 40y(C) 0% smokers	1 socket/patient single sites UJ; I/C Ca, Ct, Rf, Rr *	NR 400 *g*/12′	Flapless/SeH 10 C: blood clot 10 T: 3 L‐PRF pl *to fill*	T gave: = horizontal ABR after 4 m (*30%* vs. *30%*) **< vertical ABR after 4 m** (*19%* vs. *25%*) = width loss after 4 m measured on casts (*2.9* vs. *3.0 mm*)
Wang et al., 2022^67^ L	RCT split‐mouth 5 m L/L/L/L/L	F = 9/M = 9 mean age = 42y 0% smokers	2 sockets/patient single sites UJ & LJ; I/C/Pr **Ca**, Or, Rr	IntraSpin 408 *g*/12′	Flapless/SeH 18 C: blood clot 18 T: >1 L‐PRF pl *to fill*	T gave: = soft tissue healing (*Modified HI from Mozatti* et al., *2014*) = buccal soft tissue changes (*62* vs. *63 mm* ^*3*^) = horizontal ABR (*4.6* vs. *4.5 mm*) = vertical ABR (buccal site: *0.7* vs. *0.5 mm*)
Abad et al., 2023^68^ S	RCT parallel 4 m L/L/S/L/S	F = 14/M = 13 mean age = 58y 0% smokers *> sites buccal plate loss in T*	1 socket/patient single sites UJ & LJ; I/C/Pr **Tf**, Ca, Rer, En	Intra‐Spin 2700 rpm/12′	T: mini flap/SeH C: flapless/SeH 13 C: blood clot 14 T: L‐PRF, 2–5 pL *to fill &* 1 m *to cover*	T gave: = pain, = inflammation (VAS at w 1) = horizontal ABR: HW^1^ (*6.4* vs. *6.7 mm*), HW^3^ (*3.0* vs. *2.3 mm*), HW^5^ (*1.9* vs. *1.7 mm*) = vertical ABR: buccally (*1.9* vs. *2.7 mm*), lingually (*1.8* vs. *1.7 mm*) = vertical ridge contour reduction (*1.8* vs. *0.9 mm*) = volume loss (*77* vs. *65 mm* ^*3*^)
Al Kassar & Heshmeh 2023^69^ **H**	RCT split‐mouth 4 m H/L/H/L/S	*n* = 20 age = 18‐60y 0% smokers	2 sockets/patient single sites NR; Pr Ur, PD excl	EBA 20 2700 rpm/12′	Flapless/SeH 20 C: blood clot 20 T: 2 L‐PRF cl *to fill*	T gave: **< vertical ABR (buccal plate) after 4 m** (*0.5* vs. *1.7 mm*) **< vertical ABR (lingual plate) after 4 m** (*0.5* vs. *1.9 mm*) = horizontal ABR at crest level (*1.31* vs. *2.17 mm*) **< horizontal ABR 6 mm sub‐crestal** (*0.3* vs. *1.2 mm*)
Assadi et al., 2023^70^ S	RCT split‐mouth 2 m L/L/L/L/S	F = 21/M = 18 mean age = 42y smoker = NR	2 sockets/patient single sites UJ; I/C NR	MF200 # rpm for #′	Flapless/SeH 39 C: blood clot 39 T: ≥ 2 CGF cl *to fill*	T gave: **< pain d 2, 3, 4** **improved soft tissue healing at d 7** (*Modified HI from Mozatti* et al., *2014*) **< horizontal ABR at 1/3/5 mm after 2 m** **> buccolingual horizontal width** (*5.5* vs. *4.2 mm*) **after 2 m** **> mesiodistal horizontal width** (*5.9* vs. *4.4 mm*) **after 2 m**
Jouliq et al., 2023^71^ *2 of 4 arms* **H**	RCT parallel 6 m H/S/S/L/H	*n* = 40 age = 18‐40y smokers=NR	1 socket/patient single sites LJ; Mo Ur, PD excl	NR NR	Flapless/SeH 10 C: blood clot 10 T: 2 L‐PRF cl *2 to fill*	T gave: **< pain d 0, 2,** = edema **< horizontal ABR** (*15* vs. *28%*) = vertical ABR (*9* vs. *10%*)

*Note*: Data in bold indicate statistical significance (the first number represents the test group, the second the control group).

Abbreviations: NR, not reported; Study design: RCT, randomized clinical trial; follow‐up: w, weeks; m, months; Subjects: *n*, number; F, female; M, male; Extraction: UJ, upper jaw; LJ, lower jaw; I, incisor; C, canine; Pr, premolar; M, molar, reason for extraction (bold = most frequently, in order of decreasing importance, * no ranking): BL, bone loss; Ca, caries; Ct, cracket tooth; En, endodontic pathology; Im, implant treatment; PD excl, no periodontal disease; Oi, occlusal interference; Or, orthodontics; PD, periodontal disease; Rer, residual root; Rf, root fracture; Rr, root resorption; Tf, tooth fracture; Ups, unfavorable prosthetic support; Ur, unrestorable; Centrifugation: g, g‐force; rpm, revolutions per minute; min, minutes; Treatment: PrH, primary healing; SeH, secondary healing, c, control group; T, test group, (..), number patients/sites, PRP, platelet rich plasma; PRGF, plasma rich in growth factors; L‐PRF, leukocyte‐ and platelet rich fibrin, (acronym as mentioned in the paper: A, advanced; CGF, concentrated growth factor); cl, clot; pl, plug; m, membrane.

**TABLE 2:** Soft and hard tissue data from RCT comparing an alveolar ridge preservation using an APCs as sole biomaterial versus and other biomaterials.

Article	Study	Subjects	Extraction	Centrifugation	Treatment	Outcome parameters
	Design	Gender	Single/multiple	Device	Flap/flapless; PrH/SeH	Data in bold = statistically significant
	Follow up	Age	Tooth location: *UJ/LJ; I/C/Pr/Mo*	rpm or g/min	C = control	
Overall bias	Bias risk/domain	% Smokers	Reason for extraction	Coagulation factors	T = test	soft tissue/hard tissue healing
***A. PRP***						
No papers available						
***B. PRGF***						
Stumbras et al., 2020^45^ *3 of 4 arms* S	RCT parallel 3 m L/L/L/L/S	F = 26/M = 14 mean age = 49y 12% smokers	1 socket/patient single sites LJ; I/C **Ca**, Rf, En, PD	BTI System NR *calcium chloride*	Flapless/SeH 10 C_1_: BBM/CM 10 C_2_: FDBA/CM 10 T: 2 PRGF cl *to fill & cover with fibrin membrane*	T gave: **higher relative proportion of bone vs. C**_**1**_ **and C**_**2**_ (*76* vs. *20* vs. *7%*) **lower relative proportion of non‐mineralized tissue vs. to C**_**1**_ (*25* vs. *35* vs. *54%*)
Stumbras et al., 2021^46^ *3 of 4 arms* S	Same	Same	Same	Same	Same	T gave: = horizontal ABR at HW^1^/HW^3^/HW^5^: (*T: 1.3/0.2/0.0* vs. *C*_*1*_: *0.7/0.3/0.2 mm* vs. *C*_*2*_: *1.5/1.2/0.7 mm*) = vertical ABR (*T: 0.5* vs. *C*_*1*_ *: 0.3* vs. *C*_*2*_ *: 0.7 mm*)
***C. L‐PRF family***						
Giudice et al., 2019^49^ *3 of 4 arms* L	RCT split‐mouth 2 w L/L/L/L/L	F = 12/M = 28 mean age = 61y 38% smokers	4 sockets/patient with antiplatelets single sites NR; I/C/Pr/Mo NR	IntraSpin 2700 rpm/18′	Flap or flapless SeH 40 C: HP plug 40 T_1_: 2 L‐PRF pl *to fill* 40 T_2_: 2 A‐PRF pl *to fill*	T gave: = soft tissue healing at d 7 and w 2 = post‐extraction bleeding
Ivanova et al., 2019^57^ *2 of 3 arms* **H**	RCT parallel 4 m H/S/L/L/S	F = 31/M = 29 mean age = 42y some light smokers	1–2 socket(s)/patient single sites NR; NR NR	PRF DUO 1300 rpm/8′	Flap/PrH 28 C: FDBA 23 T: 2 L‐PRF m *to fill*	T gave: = horizontal ABR (*1.5* vs. *1.2 mm*) = vertical ABR (*0.9* vs. *0.9 mm*) = % vital bone formation (*60* vs. *66%*) = % connective tissue (*29* vs. *24%*)
Azangookhiavi et al., 2020^72^ S	RCT parallel 12 w S/L/S/L/S	F = 20/M = 12 mean age = 34y smokers = NR	1 socket/patient single sites UJ & LJ; I/C/Pr En, Rf, Ur *	NR 2700 rpm/12′	Flapless/SeH 16 C: FDBA + mucosal graft 16 T: L‐PRF m, 1 *to fill &* 1 *to cover*	T gave: = healing and maturation of soft tissue at 2 w (*clinical evaluation of soft tissue healing and maturation*) = horizontal ABR (*2.1* vs. *1.5 mm*) = vertical ABR (*0.8* vs. *0.2 mm*)
Alrayyes et al., 2022^73^ *2 of 4 arms* S	RCT parallel 4 w S/L/L/L/L	M = 18 mean age = 38y 100% *heavy smokers*	≥1 socket(s)/patient single sites UJ; Mo Ca, En, Tf *, PD excl	NR 1300 rpm/14′	Flapless/SeH NR C: FDBA/CM NR T: 1 A‐PRF m *to fill*	T gave: **improved soft tissue healing at d 10, 21 and 28** (*HI index Landry* et al., *1988*) **> soft tissue closure**
Rodrigues et al., 2023^66^ *3 of 4 arms* S	RCT parallel 4 m S/S/S/L/L	F = 18/M = 12 mean age = 42y/44y/41y:T/C_1/_C_2_ 0% smokers	1 socket/patient single sites UJ; I/C Ca, Ct, Rf, Rr *	NR 400 *g*/12′	Flapless/SeH 10 C_1_: XG + FGG 10 C_2_: d‐PTFE M (*3w*) 10 T: 3 L‐PRF pl *to fill*	T gave: **> horizontal ABR (in %) after 4 m** (*30* vs. *13 for C*_*1*_, *and 15 for C*_*2*_) **> horizontal ABR (in mm) after 4 m** (*2.9* vs. *1.0 for C1*, *and 0.9 for C*_*2*_) **> vertical ABR (in mm) after 4 m** (*2.7* vs. *1.0 for C*_*1*_, *and 1.7 for C*_*2*_)
Alrayyes et al., 2023^74^ *3 of 4 arms* L	RCT parallel 6 m L/L/L/L/L	M = 18 mean age = 38y 100% *heavy smokers*	≥1 socket(s)/patient single sites UJ; Mo Ca, En, Tf *, PD excl	NR 1300 rpm/14′	NR 10 C_1_: RCP plug 10 C_2_: FDBA/CM 10 T: *NR* A‐PRF m *to fill*	T gave: = horizontal ABR (data for respectively T, C_1_, C_2_): HW^1^ (*0.7* vs. *0.9* vs. *1.3 mm*), HW^3^ (*0.9* vs. *1.1* vs. *1.5*), HW^5^ (*0.8* vs. *1.3* vs. *1.7 mm*) = vertical ABR (*0.9* vs. *2.1* vs. *1.3 mm*)
Jouliq et al., 2023^71^ *2 of 4 arms* **H**	RCT parallel 6 m H/S/S/L/H	*n* = 40 age = 18‐40y NR	1 socket/patient single sites LJ; Mo Ur, PD excl	NR NR	Flapless/SeH 10 C: beta‐TCP 10 T: 2 L‐PRF cl *to fill*	T gave: = vertical ABR (*9* vs. *7%*) = horizontal ABR (*15* vs. *11%*)

*Note*: Data in bold indicate statistical significance (first number represents test group, the second the control group).

Abbreviations: NR, not reported; Study design: RCT, randomized clinical trial, follow‐up: w, weeks, m, months; Subjects: *n*, number; F, female, M, male; Extraction: UJ, upper jaw; LJ, lower jaw; I, incisor; C, canine; Pr, premolar; M, molar, reason for extraction (bold = most frequently, in order of decreasing importance, * no ranking): Ca, caries; Ct, cracket tooth; En, endodontic pathology; PD excl, no periodontal disease; PD, periodontal disease; Rf, root fracture; Rr, root resorption; Tf, tooth fracture; Ur, unrestorable; Centrifugation: g, g‐force; rpm, revolutions per minute; min, minutes; Treatment: PrH, primary healing; SeH, secondary healing; c, control group; T, test group, (..), number patients/sites; PRP, platelet rich plasma; PRGF, plasma rich in growth factors; L‐PRF, leukocyte‐ and platelet rich fibrin, (acronym as mentioned in the paper: A, advanced; CGF, concentrated growth factor); cl, clot; pl, plug; m, membrane; BBM, bovine bone mineral; FDBA, freeze‐dried bone allograft; CM, collagen membrane; RCP, resorbable collagen plug; HP, haemostatic plug; TCP, tricalcium phosphate; d‐PTFE M, dens polytetrafluoroethylene membrane; XG, xenograft; FGG, free gingival graft; SVS, simvastatin.

### 3.4 APC versus unassisted healing

In total, 31 RCTs (32 papers) compared the outcome of an ARP utilizing an APC with unassisted healing (non‐third molars) (Table 1). One study reported on the use of PRP, five on PRGF (6 papers), and 25 on L‐PRF. The majority of papers (*n* = 25) followed the socket healing for ≥8 weeks, while seven papers only took into consideration a short‐term follow‐up (≤3 weeks).

#### 3.4.1 PRP

Alissa et al.^40^ applied a PRP gel in the extraction socket and coronally advanced the gingiva for primary closure (Table 1A). Compared to unassisted healing, they observed in favor of PRP: more trabecular bone volume (43% vs. 32%), improved soft tissue healing (4.1 vs. 3.1 as healing index), and superior PROMs during the first days (e.g., less pain and lower analgesic consumption). Data on alveolar bone resorption (ABR) were not available (Table 1A).

#### 3.4.2 PRGF

With a flapless technique and healing by secondary intention, five RCTs (6 publications) evaluated the advantages of PRGF during ARP in comparison to unassisted healing (Table 1B).^41^, ^42^, ^43^, ^44^, ^45^, ^46^ In all studies, PRGF was used as socket filler (gel or clot), and in four of them, the entrance to the socket was sealed with an autogenous fibrin membrane.

##### Alveolar ridge dimensional changes

Five papers focused on alveolar bone healing. ABR was examined in two articles. While Stumbras et al.^46^ did not find any statistically significant differences, whether in horizontal or vertical dimension alterations, Anitua et al.^43^ observed significantly greater socket bone fill (97% vs. 46%).

Four papers assessed the quality of the regenerated bone. Using both radiographic and histomorphometric parameters, Farina and colleagues were unable to find any benefit following the application of PRGF.^41^ In contrast, three other studies discovered statistically significant improvements in features such as bone density (450 vs. 318; and 648 vs. 500 Hounsfield units)^43^, ^44^; and/or the relative proportion of bone (63% vs. 36%; 76% vs.46%) in favor of PRGF.^43^, ^45^

##### Soft tissue healing/postoperative pain

One short‐term study reported a better soft tissue healing and a significant decrease in residual socket volume after the application of PRGF.^42^ Two articles looked at pain levels after therapy. Mozzati et al.^42^ saw no difference, while Anitua et al.^43^ reported significantly less pain when using PRGF (Table 1B).

#### 3.4.3 L‐PRF

Twenty‐five RCTs explored the benefits of using L‐PRF as a single biomaterial for ARP, six with a short follow‐up of ≤21 days,^47^, ^48^, ^49^, ^50^, ^51^, ^52^ and 19 with a follow‐up of 2–6 months (Table 1C).^53^, ^54^, ^55^, ^56^, ^57^, ^58^, ^59^, ^60^, ^61^, ^62^, ^63^, ^64^, ^65^, ^66^, ^67^, ^68^, ^69^, ^70^, ^71^ The studies revealed a large heterogeneity in various aspects:

- In the majority of studies, single sites were considered, but in two studies, multiple sites adjacent to each other were included^47^, ^61^;
- Most studies chose a flapless approach (*n* = 19), but in six, either a flap had been raised,^57^, ^63^ a mini flap,^68^ or a mixture of both concepts was allowed^49^, ^51^, ^53^;
- Most surgical protocols anticipated healing by secondary intention, but in two studies, healing by primary intention (flap closure) was chosen,^57^, ^63^ and one study used both healing options;^51^
- The type of L‐PRF matrix to fill the extraction socket was different: Seven used clots, 10 membranes, and eight plugs;
- The amount of L‐PRF inserted into the extraction socket was different: In eight studies, only one matrix was inserted, while 17 trials used at least two clots/plugs/membranes to fill the socket;
- In some studies (*n* = 6), an L‐PRF membrane was also used as a barrier membrane to cover and seal the socket^53^, ^54^, ^60^, ^61^, ^64^, ^68^;
- The majority of studies (*n* = 12) included teeth from both the maxilla and mandible, four only from the maxilla, one only from the mandible, and eight studies did not report this information;
- Not all studies included the same tooth types; four selected only incisors or canines; two selected only premolars; one selected only molars; six included incisors, canines, and premolars; five included premolars and molars; one included canines, premolars, and molars; and two included all tooth types (except 3rd molars), but four studies did not report the tooth type.

##### Alveolar ridge dimensional changes

The impact on hard tissue healing of applying L‐PRF in the extraction socket versus unassisted healing was investigated in 19 RCTs.

Fourteen papers presented data on alveolar bone resorption (ABR), enrolling 383 patients and a total of 544 sockets using a variety of parameters. Eleven studies reported significantly less ABR in the horizontal and vertical direction in favor of L‐PRF (e.g., using proportional horizontal ABR data: 75% less^53^; 56% less^54^; 47% less^55^; 38% less^57^; 33% less^59^; 61% less^60^; 55% less^64^; 75% less^69^; 88% less^70^; and 46% less^71^), or significantly more socket fill (89 vs. 80%^55^; 95 vs. 63%^54^; 85 vs. 68%^61^). One paper observed a superiority for L‐PRF only in the reduction of vertical height resorption.^66^ Two papers, however, failed to find any statistically significant differences.^67^, ^68^

Ten papers discussed the bone quality.^53^, ^56^, ^57^, ^58^, ^59^, ^60^, ^61^, ^62^, ^63^, ^64^ Two research groups found no difference between unassisted healing and the application of L‐PRF (histology^56^; density^59^). The other eight papers reported several statistically significant differences, all in favor of L‐PRF, including a higher relative proportion of bone (61% vs. 37%^57^; 56% vs. 40%^60^; 48% vs. 35%^61^; 54% vs. 41%^62^; 61% vs. 39%^63^; 60% vs. 50%^64^), higher intrinsic mechanical properties,^53^ a higher bone density (1121 vs. 936 mg/cm^3^ ^64^), or a higher osseous fractal dimension (1.33 vs. 1.24^58^); for more details, see Table 1C.

##### Soft tissue healing/postoperative pain

Eleven papers looked at soft tissue healing.^47^, ^48^, ^49^, ^50^, ^51^, ^52^, ^58^, ^64^, ^65^, ^67^, ^70^ Nine reported superior soft tissue healing (typically measured by a healing index) and/or faster wound epithelialization and/or wound closure in favor of L‐PRF, while two found no differences [Giudice et al.^49^ (with patients taking antiplatelets), Wang et al.^67^].

Eleven studies analyzed the effect of L‐PRF on pain following extraction; with eight reporting a statistically significant pain reduction, particularly during the first days,^47^, ^50^, ^51^, ^52^, ^54^, ^58^, ^70^, ^71^ but in three, the pain reduction did not reach statistical significance.^48^, ^65^, ^68^

The post‐extraction consumption of analgesics was recorded in three studies,^50^, ^51^, ^58^ two reporting a statistical reduction when using L‐PRF.^50^, ^51^

#### 3.4.4 Meta‐analyses

**FIGURE 4:**
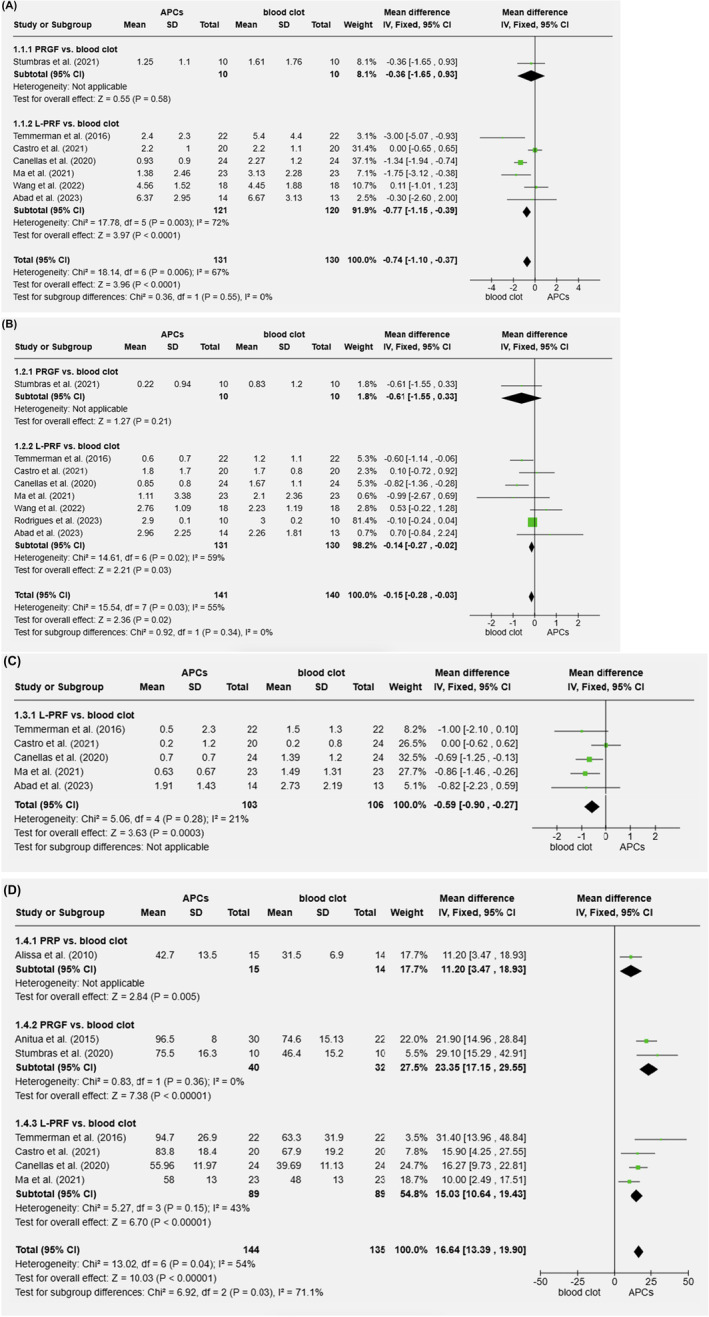
Forest plots illustrating the impact of APC on alveolar ridge dimensional changes after tooth extraction compared to unassisted healing. (A) Horizontal width reduction 1 mm below the crest. (B) Horizontal width reduction 2–3 mm below the crest. (C) Reduction of the vertical height of the buccal bone. (D) Percentage of socket fill.

Meta‐analyses were performed with papers using the “same parameters” measured at “the same location,” including standard deviations, and with follow‐up periods ranging from 2.5 to 5 months (Figure 4).^40^, ^43^, ^45^, ^46^, ^54^, ^60^, ^61^, ^64^, ^66^, ^67^, ^68^ Papers with a high risk of bias were not included.

One paper for PRGF, six for L‐PRF, and no paper for PRP compared the amount of horizontal alveolar bone resorption assessed at 1 mm apically to the crest in Figure 4A. There was no statistically significant advantage for PRGF. Despite significant variability across the studies, L‐PRF significantly reduced the resorption when compared to unassisted healing (*p* < 0.0001, mean difference − 0.8 mm, 95% CI: −1.2 to −0.4). The parameter in Figure 4B is the same, but now measured 2–3 mm apically to the crest. The required data for this parameter were only provided by one paper on PRGF and seven on L‐PRF. The application of L‐PRF led to considerably less resorption (*p* = 0.03, mean difference − 0.1 mm, 95% CI: −0.3 to 0.0), again with significant variability between the experiments. PRGF did not reveal a meaningful difference.

The changes in the buccal bone plate's vertical height are displayed in Figure 4C. Once again, L‐PRF sites exhibit significantly less resorption (*p* = 0.0003, mean difference −0.6 mm, 95% CI: −0.9 to −0.3). For PRP or PRGF, there was no data available.

Figure 4D compiles the data on the percentage of socket fill; it includes four papers on L‐PRF, two on PRGF, and one on PRP. For all three APC, there was a statistically significant increase in percentage socket fill: 11.2% for PRP, 23.4% for PRGF, and 15.0% for L‐PRF (with significant heterogeneity across the three APC types, but not within each type).

### 3.5 APC versus other biomaterials

A total of seven RCTs (9 papers) evaluated the use of an APC with another biomaterial during ARP.^45^, ^46^, ^49^, ^57^, ^66^, ^71^, ^72^, ^73^, ^74^ Only one study (2 papers) applied PRGF, and six studies (7 papers) used L‐PRF, whereas we were unable to identify any papers that employed PRP for comparison. With the exception of two studies that had a short follow‐up (2 and 4 weeks), the follow‐up was relatively long (3–6 months) (Table 2).

#### 3.5.1 PRP

There were no studies available.

#### 3.5.2 PRGF

Stumbras et al. compared the use of PRGF as a single socket filler with bovine bone mineral (BBM) or freeze‐dried bone allograft (FDBA), the latter two in combination with a socket sealing [a collagen membrane (CM)]. Histology revealed more newly formed bone (76% vs. 20% vs. 7%) in the PRGF group compared to the BBM/CM or FDBA/CM groups.^45^, ^46^ The BBM/CM group showed the least horizontal and vertical alveolar bone resorption, although the differences were not statistically significant, neither from the PRGF group nor from the FDBA/CM group (Table 2B).

#### 3.5.3 L‐PRF

Seven papers compared L‐PRF as the sole biomaterial with another biomaterial, two over a short follow‐up period (≤28 days),^49^, ^73^ and five over a longer time interval (3–6 months).^57^, ^66^, ^71^, ^72^, ^74^

Giudice et al.^49^ compared the soft tissue healing after 2 weeks between a hemostatic plug, an L‐PRF plug, and an A‐PRF plug in patients under antiplatelet medication and found a similar soft tissue healing. Alrayyes et al. compared the soft tissue healing (up to 4 weeks) after an ARP procedure using either an FBDA bone substitute combined with a collagen membrane or a single A‐PRF membrane in the socket. The latter was superior in soft tissue healing and soft tissue closure.^73^

Five studies compared the dimensional changes of the alveolar ridge between an L‐PRF application (as a single substitute to fill the socket, and in one study, also to cover the socket as a fibrin membrane) with various bone substitutes [FDBA in three studies, xenograft in one study, and β‐TCP (beta tricalcium phosphate) in another study], sometimes in combination with a barrier membrane or a soft tissue graft to seal the entrance to the socket, and occasionally a healing by primary intention. Ivanova et al. compared the outcome of an FDBA application with that of an L‐PRF socket fill, both via healing by primary intention. They did not observe any significant differences, neither in ABR nor in bone quality.^57^ Azangookhiavi et al.^72^ compared FDBA covered with a mucosal graft to L‐PRF membranes (1 to fill, 1 to cover), and they discovered that the L‐PRF group had greater horizontal (2.1 vs. 1.5 mm) and vertical (0.8 vs. 0.2 mm) resorption; however, despite having 16 patients in each group, this difference was not statistically significant. Alrayyes et al. conducted a comparison between (i) socket grafting using A‐PRF membranes with (ii) a socket fill using FDBA and a collagen membrane in one group and (iii) the placement of a resorbable collagen plug in another group.^74^ There were no statistically significant differences between the three treatment approaches. The team of Rodrigues compared three ideas: a sole application of three L‐PRF plugs, a socket fill with a xenograft sealed with a free gingival graft, or the application of a d‐PTFE membrane for 3 weeks.^66^ The sole L‐PRF treatment was significantly inferior in reducing horizontal and vertical alveolar bone resorption. A socket fill using beta‐TCP or L‐PRF was compared by Jouliq and colleagues, both with a healing by secondary intention. The percentages of horizontal and vertical ABR were the same for both treatments (Table 2C).^71^

## 4 DISCUSSION

### 4.1 Risk of bias

The overall risk of bias in the included papers is high, with 8/35 papers at low risk, 16/35 at moderate risk, but 11/35 at high risk of bias (2/6 papers on PRGF and 9/29 on L‐PRF). The primary causes for this were inadequacies in the randomization procedure (*n* = 6), the blinding strategy pertaining to participants, surgeons, and personnel (*n* = 5), as well as toward outcome assessment (*n* = 4). The high scores for bias might be explained by the fact that both examiners have been very strict in their judgment. Furthermore, because most studies did not carry out or submit the randomized clinical trial registration, they were categorized as having “some concerns” in the “selection bias of reported results domain.”

### 4.2 APC versus unassisted healing

#### 4.2.1 Alveolar ridge dimensional changes

There are surprisingly few papers investigating alveolar bone resorption following the application of PRGF (*n* = 2) or PRP (*n* = 0). The paper by Stumbras et al.^46^ did not observe any significant benefit on this parameter, possibly as a result of the small patient population. However, Anitua and colleagues^43^ reported that PRGF had a statistically significant greater bone fill in the extraction socket.

Fourteen papers compared the alveolar ridge resorption between unassisted healing and a socket fill with L‐PRF, with 12 reporting significant advantages in favor of L‐PRF,^53^, ^54^, ^55^, ^57^, ^59^, ^60^, ^61^, ^64^, ^66^, ^69^, ^70^, ^71^ but with two papers indicating no benefit of L‐PRF use.^67^, ^68^ The statistical superiority demonstrated by the meta‐analyses supports the beneficial effect of L‐PRF on the reduction in alveolar bone resorption, despite the inclusion of some papers that raise concerns, see below.^61^, ^67^, ^68^

#### 4.2.2 Speculation on impact of therapeutic approach when applying L‐PRF during ARP

The data above on the benefits of L‐PRF show some inconsistencies, but the authors feel, based on their own experience, that the majority of these variances may be attributed to variations in the surgical technique and case selection. Table 3 enumerates these features.

**TABLE 3:** Impact of treatment strategy on the benefit of L‐PRF as a single biomaterial during ARP.

Author	Significant impact		L‐PRF application			Flap	Tooth type	Other remarks
*n* = 13	Horizontal	Vertical	Socket sealing	PrH/SeH	Number as fill	Flap or flapless		
Hauser et al 2013 arm 1	+	NR	y	SeH	4 m	Flapless	UJ & LJ; Pr	
Hauser et al 2013 arm 2	−	NR	y	PrH	4 m	Flap	UJ & LJ; Pr	
Temmerman et al., 2016	+	+	y	SeH	2–5 m	Flapless	UJ & LJ; I/C/Pr	
Alzahrani et al., 2017 (H)	+	NR	n	SeH	2 m	Flapless	NR; NR	
Ivanova et al., 2019 (H)	+	+	y	PrH	2 m	Flap	NR; NR	
Alsayed et al., 2020 (H)	+	+	n	SeH	2 cl	Flapless	NR; NR	
Canellas et al., 2020	+	+	y	SeH	4 pl	Flapless	UJ & LJ; I/C/Pr	
Castro et al., 2021	Only + for socket fill		y	SeH	2–3 m	Flapless	UJ; I/C	Multiple sites + removable denture
Ma et al., 2021	+	+	y	SeH	1 cl	Flapless	UJ & LJ; Pr/Mo	
Rodrigues et al., 2023	−	+	n	SeH	3 pl	Flapless	UJ; I/C	
Wang et al., 2022	**−**	**−**	n	SeH	>1 pl	Flapless	UJ & LJ; I/C/Pr	Nearly only premolars
Abad et al., 2023	**−**	**−**	y	SeH	2–5 pl	Mini‐flap	UJ & LJ; I/C/Pr	Case selection/experience
Al Kassar & Heshmeh 2023 (H)	+	+	n	SeH	2 cl	Flapless	NR; Pr	
Assadi et al., 2023	+	NR	n	SeH	≥2 cl	Flapless	UJ; I/C	
Jouliq et al., 2023 (H)	+	−	n	SeH	2 cl	Flapless	LJ; Mo	

*Note*: Colors: Green when significant benefits were observed in favor of L‐PRF; orange indicated less favorable surgical approach/conditions.

In one study by Castro et al.,^61^ *multiple extraction* sockets neighboring each other were included, and the patients were wearing a denture during the healing period. It is indeed well known that when ARP is conducted in several neighboring sockets, the reduction in alveolar ridge resorption is less.^6^ Moreover, the mechanical pressure transferred continuously and/or intermittently through the full immediate *removable prosthesis* has been considered one of the causative factors for bone resorption in denture‐supporting tissues.^75^, ^76^

A second prerequisite is a *minimally invasive* surgical approach. The study by Abad et al.,^68^ but not by Ivanova et al.,^57^ indeed shows that raising a flap diminishes the positive impact of a solitary application of L‐PRF, as previously demonstrated by Hauser and colleagues^53^ comparing a flap and flapless ARP with each other (using L‐PRF).

The integrity of the bone walls also influences the feasibility of bone regeneration. In the study by Abad et al.,^68^ the large difference in integrity (10/13 intact buccal bone plates in the control group vs. 3/14 in the test group) might explain why they did not reach a statistical difference between L‐PRF sites and unassisted healing, besides the diversity in clinical experience between operators.

When considering *socket sealing*, 8/18 studies correlated a “sealing” or “no sealing” correctly with, respectively, a “strong” and “weak” benefit. The sealing of the entrance to the socket with an L‐PRF membrane (running over the bony borders) will indeed reduce the ingrowth of soft tissue into the socket that could hinder new bone formation. This might explain the lack of benefits for L‐PRF reported by Rodrigues et al.^66^ and Wang et al.^67^

When the *number of L‐PRF matrices* applied within the socket is considered, 10/13 studies correlated a “several” or “only one” matrix correctly with, respectively, a “strong” and “weak” benefit. From the 13 studies in which more than one L‐PRF matrix was applied in single sockets (with the inclusion of comparable socket conditions), excluding Castro et al.^61^ (multiple sites and denture wearing), Abad et al.^68^ (unequal conditions in test and control, questionable experience), Rodriques et al.^66^ and Wang et al.^67^ (no socket sealing), all remaining nine studies obtained a statistically significant reduction of the ABR.^53^, ^54^, ^55^, ^57^, ^59^, ^60^, ^69^, ^70^, ^71^

When applying *the protocol* described by Temmerman et al.^54^ and Canellas et al.^60^ (the application of 2–5 L‐PRF membranes/plugs, a socket sealing with an L‐PRF membrane, a flapless approach, a single extraction site, no pressure on the healing socket, follow‐up of 3 months), the following clinical outcome was reached: less horizontal ABR (a mean reduction of 2.7 mm compared to unassisted healing) and less vertical height ABR (a mean reduction of 0.8 mm) in favor of L‐PRF.^54^, ^60^ These reductions are quite similar to what has been concluded in a recent consensus report (European Workshop in Periodontology) for an ARP (barrier membrane, biomaterial, or a combination of both): 1.5–2.4 mm less horizontal and 1.0–2.5 mm less mid‐buccal vertical bone resorption as compared to spontaneous healing.^34^

**FIGURE 5:**
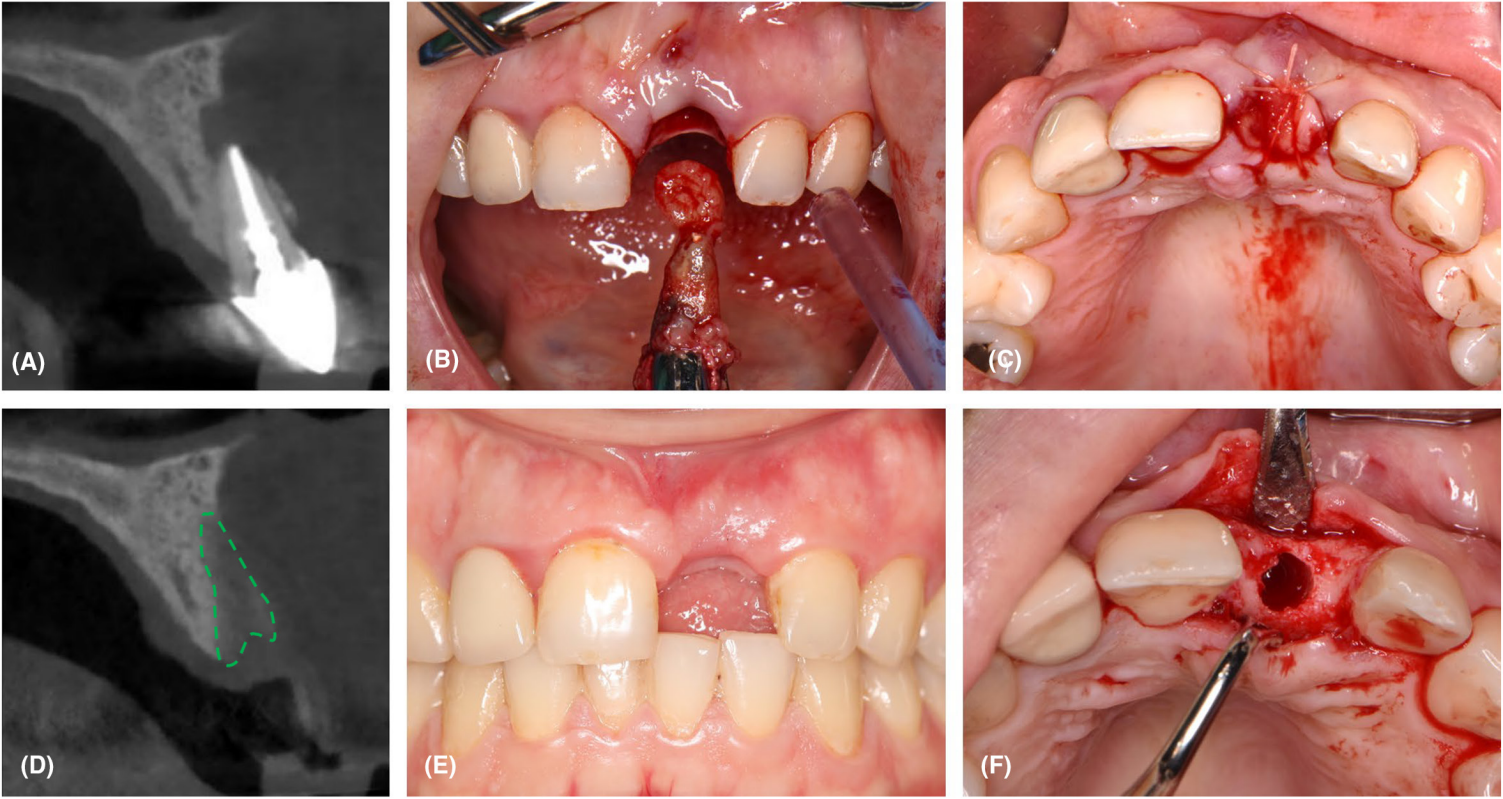
Clinical case of ARP with L‐PRF in the anterior zone. (A) Cross‐sectional CBCT image of tooth number 21, prior to extraction; (B) Extraction of tooth number 21 including the apical granuloma; (C): Application of L‐PRF membranes (2 membranes to fill and 1 double layer L‐PRF membrane to cover) inside the extraction socket of tooth number 21 and placing a positioning suture; (D) Cross‐sectional CBCT image after 3 months of healing; (E) Frontal view showing the soft tissue healing at the extraction site; (F) incisal view showing the osteotomy preparation for dental implant at site 21 (favorable horizontal and vertical ridge dimensions and good vascularization).

**FIGURE 6:**
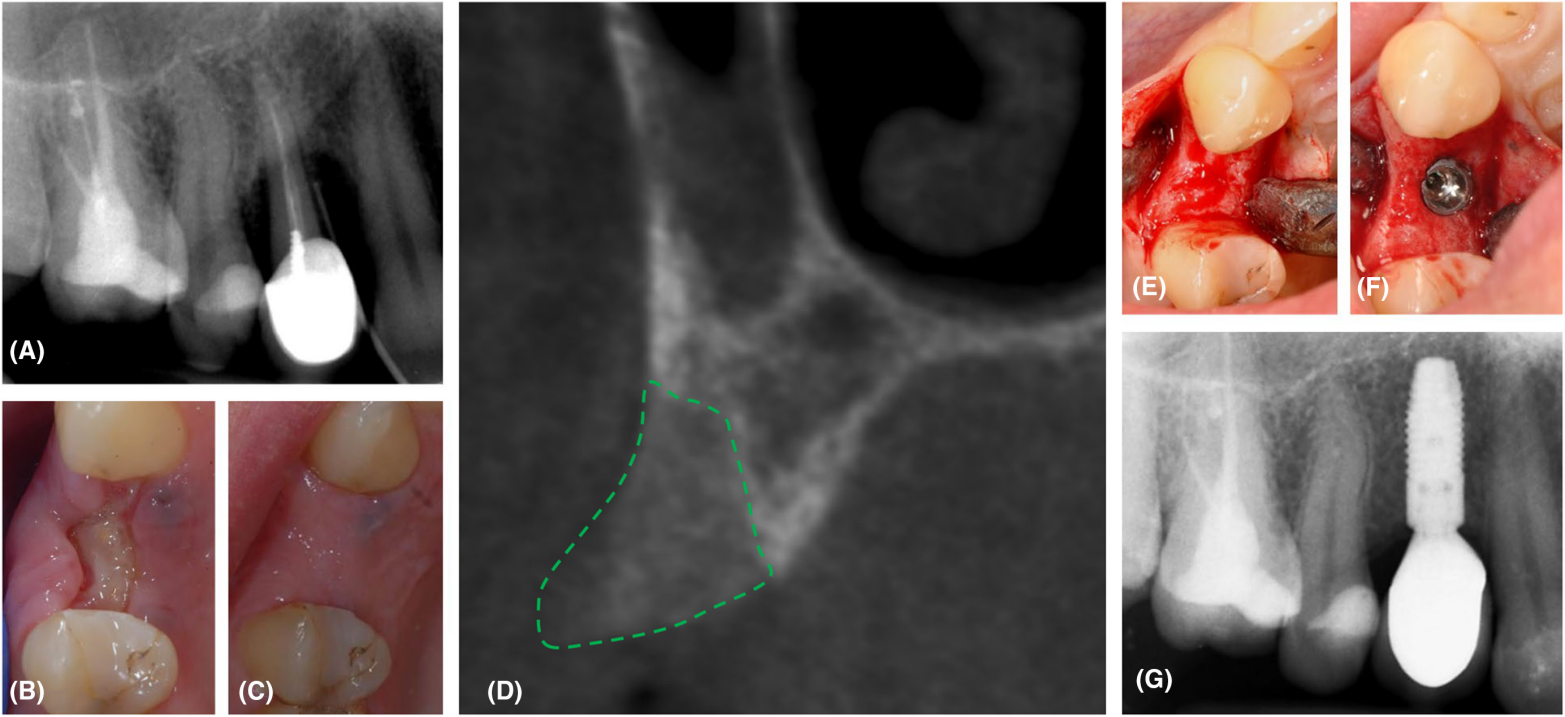
Clinical case of ARP with L‐PRF in the posterior zone. (A) The first premolar in the upper right side was extracted (loss of the entire buccal bone plate, PPD > 10 mm, endodontic pathology); (B) After 1 week, the soft tissue starts covering the top L‐PRF membrane, which is sealing the entrance to the socket; this membrane shows some pink spots (probably due to vascularization); (C) Good soft tissue healing after 3 months with minimal change in alveolar crest dimensions; (D) Cross‐sectional CBCT image in the center of the extraction socket showing optimal bone regeneration with moderate bone calcification; (E) Re‐entry after 3 months with the entire re‐generation of the buccal bone plate; (F) Insertion of a 3.6 mm diameter Astra‐Tech EV implant; (G) Intra‐oral radiograph after 7 years of loading.

Two examples (Figures 5 and 6) illustrate the Temmerman et al. protocol.^54^ In Figure 5, L‐PRF has been applied in the socket of a central incisor, with a nice healing and enough alveolar bone width to place an implant in the ideal position; the small remaining buccal concavity was filled with a soft tissue graft. Figure 6 shows the healing following the extraction of a first premolar. The socket is filled with several L‐PRF plugs, and the socket entrance is sealed with a double layer of L‐PRF membranes, which assist the soft tissue healing and permit secondary intention healing. The absence of a primary wound closure significantly simplifies the surgery since there is no longer a need to create a flap that could also impede the blood supply.

Videos on the applied surgical technique are available via the following link: https://www.l‐prf4all.com/lprfperio2000additionalvideos.

#### 4.2.3 Regenerated bone quality

Only one paper, applying PRP, examined this parameter and observed a higher proportion of trabecular bone.^40^ Four papers looked at the regenerated bone in the extraction socket filled with PRGF, with three papers showing significant benefits in favor of PRGF (higher bone density and/or the relative fraction of mineralized bone and/or the relative proportion of bone vs. connective tissue),^43^, ^44^, ^45^ which contrasts with the findings of Farina et al.,^41^ indicating no benefits without providing a clear explanation for the discrepancy.

Ten RCTs discussed the bone quality following the application of L‐PRF.^53^, ^56^, ^57^, ^58^, ^59^, ^60^, ^61^, ^62^, ^63^, ^64^ Two did not observe any difference when compared to unassisted healing (Areewong et al.,^56^ Alsayed et al.^59^), but the other eight reported several statistically significant differences, all in favor of L‐PRF, including a higher relative proportion of bone, higher intrinsic mechanical properties,^53^ higher bone density,^64^ or a higher osseous fractal dimension.^58^ Potential explanations for these contradictory observations are:

- For Areewong et al. (analyzing bone cores): the use of a single L‐PRF plug to fill the socket, no socket sealing, the short healing time (8 weeks), and the fact that the biopsies were limited to the coronal/central part of the socket [biopsies of the coronal 6 mm (2 mm in diameter); 8/36 biopsies even collected only connective tissue].^56^
- For Alsayed et al. (CBCT analyses): doubtful technique to measure the bone density (unclear which area was measured, no baseline scores, artificial Hounsfield values, …).^59^

Potential mechanisms for the positive impact of APC on hard tissue healing are summarized in detail in three other papers in this special issue.^77^, ^78^, ^79^

#### 4.2.4 Soft tissue healing

One study utilizing PRP and two employing PRGF demonstrated a positive impact on soft tissue healing as compared to unassisted healing.^40^, ^42^, ^43^ For the application of L‐PRF, nine RCTs reported a faster soft tissue healing and/or a faster wound epithelialization and/or a wound closure versus spontaneous healing^47^, ^48^, ^50^, ^51^, ^52^, ^58^, ^64^, ^65^, ^70^, but two did not observe any differences.^49^, ^67^ The latter might be explained by:

- For Guidice et al.^49^: the inclusion of only patients on antiplatelets, the applied soft tissue healing index (no reference), the absence of a socket sealing; however, the socket fill with L‐PRF (2/10) or A‐PRF plugs (1/10) did result in a significant reduction of bleeding 30 minutes after the extraction when compared to unassisted healing (8/10) or an hemostatic plug (5/10);
- For Wang et al.^67^: no clear explanation, except perhaps the absence of a socket sealing.

The beneficial effect of L‐PRF on soft tissue healing has also been observed in clinical trials examining the use of L‐PRF membranes as surgical dressing for intra‐oral wounds [e.g., after retrieving a free gingival graft or a subcutaneous connective tissue graft,^80^, ^81^, ^82^, ^83^, ^84^, ^85^, ^86^, ^87^ as well as for the protection of large wounds^88^, ^89^, ^90^, ^91^, ^92^]. This adjunctive effect is even more impressive when looking at the soft tissue healing of chronic wounds (e.g., diabetic foot, venous leg ulcer, leprosy wound) or burns after the application of L‐PRF or PRP (for more details, see Perussolo et al.,^93^ Pinto et al.^94^).

Potential mechanisms for the positive impact of APC on soft tissue healing are presented in detail in three other papers in this special issue.^77^, ^78^, ^79^

#### 4.2.5 Pain

One paper on PRP^40^ and two on PRGF ^42^, ^43^ examined their impact on pain. Only Alisa et al. and Anitua et al. observed a beneficial effect in comparison to unassisted healing.^40^, ^43^

A statistically significant decrease in pain was observed in eight out of the 11 RCTs that compared the effect of L‐PRF on post‐extraction pain with spontaneous healing, particularly in the first 3 days.^47^, ^50^, ^51^, ^52^, ^54^, ^58^, ^70^, ^71^ Moreover, two studies reported a statistical reduction in the consumption of analgesics after the use of L‐PRF.^50^, ^51^ This pain reduction was also reported in a recent systematic review of Siawasch et al. exploring the benefits of APC application following third molar extractions.^95^

The analgesic effect of APC is ascribed to: a reduction in prostaglandins (the main mediators of pain perception),^96^, ^97^ the release of growth factors and cytokines, but also to the stable fibrous network, particularly with L‐PRF, containing leukocytes, matrix proteins, and anti‐inflammatory mediators released for an extended period of time functioning throughout the inflammatory repair phase,^98^, ^99^ as well as the slow dissolution of the L‐PRF, which prevents debris impaction and protects the underlying blood clot.^100^, ^101^

### 4.3 APC versus other biomaterials

The number of papers comparing the benefits of PRP (*n* = 0), PRGF (*n* = 2,^45^, ^46^), or L‐PRF (*n* = 7,^49^, ^57^, ^66^, ^71^, ^72^, ^73^, ^74^) to another biomaterial reporting on alveolar ridge resorption is scarce. Due to the large heterogeneity in treatment concepts (bone substitute, primary vs. secondary healing, barrier membrane or not), it was simply impossible to make evidence‐based statements, even though, in only one trial, the APC was inferior to the other biomaterial in horizontal and/or vertical ABR.^66^ Perhaps the lack of differences could be related to the treatment strategies, the low number of patients enrolled in the studies, and/or the fact that the differences are indeed small.

### 4.4 Critical considerations

The present article is not exempt from limitations. The most important limitation is the large *heterogeneity* among the included studies. They showed a high degree of variability, including variation in: APC [1st and 2nd generation; their preparation (e.g., original L‐PRF vs. A‐PRF vs. CGF)], surgical technique (APC to fill the socket, to seal the socket, or both), amount of APC (e.g., number of L‐PRF clots/plugs/membranes), timing of assessment, and outcome parameters. It is also crucial to note that some studies evaluated the total change in horizontal or vertical dimensions, others distinguished between the buccal and lingual plates; some only measured at the crestal level while others measured at different levels apically from the crest, etc.

Moreover, a significant number of additional *confounders* were even not considered, such as antibiotic prescription, smoking (not a single study made a direct comparison between smokers and non‐smokers), wound protection (partial or full denture, acid edge bridge, nothing), and use of a barrier membrane.

The *tooth selection* for ARP also showed a wide variety [some only included incisors or canines, some only premolars or only molars, and others included a mixture of incisors, canines, and premolars, or premolars and molars, or even all tooth types (except 3rd molars)]. Some studies only included maxillary sites, and others only sites in the mandible. However, it is known that the pattern and extent of socket healing are influenced by the location of the extraction site (maxilla or mandible, and posterior or anterior regions). The alveolar bone resorption after tooth extraction is generally larger in the mandible than the maxilla, and more in the posterior than the anterior region.^16^, ^102^ Couso‐Queiruga et al., for example, reported a mean horizontal bone loss (in both jaws) of 3.6 mm in the posterior region versus 2.5 mm in the anterior region, as well as a mean vertical bone loss (buccally) of 1.5 mm in the molar region and 1.7 mm in the anterior region.^3^

Moreover, a thin *buccal bone morphology* is found to pronounce bone resorption^6^, ^10^, ^19^, ^103^, ^104^ making these sites (e.g., incisors and canines in the maxilla) more vulnerable for alveolar bone resorption.^12^, ^105^

The *reason* for tooth extraction can have an impact on the outcome of ARP. Teeth with a history of periodontitis, for example, show delayed socket healing, and advanced bone loss prolongs the time of osseous regeneration in the socket.^12^, ^106^, ^107^ Ben Amara et al. even reported a significant positive relationship between the degree of baseline bone loss and post‐extraction ABR.^108^ The reduced surface area of bony walls in periodontally compromised sockets will limit tissue resources with less regenerative potential.^109^

The *integrity* of the socket walls after tooth extraction also influences the outcome of ARP. An extraction socket with four intact walls represents the most favorable situation for healing, as the greater the number of walls, the more: (i) protection and stability for the clot, (ii) bone cells, and (iii) blood supply.^12^, ^110^ Sites with buccal wall defects indeed show more pronounced dimensional changes.^111^ In the paper by Abad et al., the integrity of the buccal wall was clearly lower in the L‐PRF group than the control group, a factor that might explain why no benefit could be obtained with the L‐PRF application.^68^

Also, the variety in data assessment is significant. Several methods (CBCT, intra‐oral measurements, and cast models) were used to *measure* the alveolar dimensional changes, but based on a meta‐regression and sensitivity analysis, it was found that the CBCT method is most appropriate.^22^ Cast analysis is most unreliable as the bone changes would be overexpressed.

Moreover, the *interval* between extraction and bone resorption assessment varied between 2 weeks and several months (often 6 months). We observed that it takes up to 4 months before the bone in the extraction socket becomes clearly visible on a CBCT.

Also, the *bone density* quantification showed a large variety, ranging from gray value assessments on intra‐oral radiographs to Hounsfield measurements (often artificial estimations) on CBCT.

Most papers with *histomorphometric* outcomes presented a small sample size, limiting the possibility of drawing strong conclusions.

Other significant limitations are a lack of clinical *subgrouping* (e.g., according to the initial condition of the socket walls, the patients' individual genotypical alveolar bone morphology,^112^ and the high risk of *bias*, mainly due to randomization, causes a low certainty of evidence).

## 5 CONCLUSION

Within the limitations of this review, it can be recommended to use APC for ARP procedures to improve soft and hard tissue healing in order to reduce post‐extraction alveolar ridge resorption. Due to more extensive scientific evidence and a more simplified preparation protocol, the use of L‐PRF might be preferred. The ideal approach for ARP with L‐PRF would consist of grafting the alveolar socket with more than one L‐PRF membrane/plug, followed by a socket sealing with an additional L‐PRF membrane, and a flapless approach. The small number of studies comparing an APC with other biomaterials rendered strong statements impossible.

**Table:** 

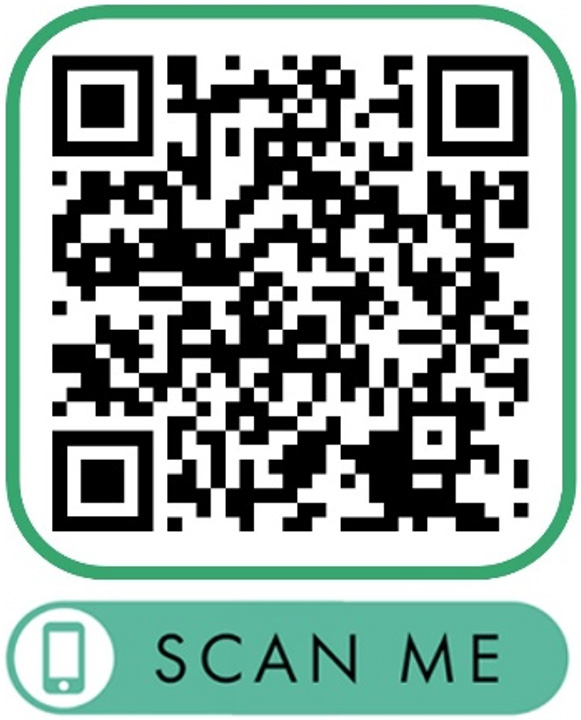	For more information on the use of L‐PRF matrices in alveolar ridge preservation, you may wish to visit a webpage from Marc Quirynen with short videos summarizing the procedure. Just scan this QR code. You can also use this URL: https://www.l‐prf4all.com/lprfperio2000additionalvideos

## CONFLICT OF INTEREST STATEMENT

All authors declare that they have no conflict of interest in relation to this chapter. The Department of Periodontology at the KU Leuven has received research support from different implant companies including Dentsply Sirona, Straumann and Henry Schein. Drs. Yu received support from the China Scholarship Council (File No. 202206170027).

## Data Availability

Data sharing is not applicable; no new data generated.
